# Across the deserts and sea: inter-individual variation in migration routes of south-central European barn swallows (*Hirundo rustica*)

**DOI:** 10.1186/s40462-022-00352-3

**Published:** 2022-11-22

**Authors:** Mattia Pancerasa, Roberto Ambrosini, Andrea Romano, Diego Rubolini, David W. Winkler, Renato Casagrandi

**Affiliations:** 1 Dipartimento di Elettronica, Informazione e Bioingegneria, Politecnico Di Milano, Via Ponzio 34/5, 20131 Milan, Italy; 2 Dipartimento di Scienze e Politiche Ambientali, Università degli Studi di Milano, Via Celoria 26, 20133 Milan, Italy; 3 Istituto di Ricerca sulle Acque, IRSA-CNR, Via del Mulino 19, 20861 Brugherio, MB Italy; 4 SABER Consulting, P.O. Box 293, Monterey, CA 93942 USA

**Keywords:** Long-distance movement, Carry-over effects, Ecological barriers crossing, Light-level geolocators, Loop migration

## Abstract

**Background:**

The spatiotemporal organization of migratory routes of long-distance migrants results from trade-offs between minimizing the journey length and *en route* risk of migration-related mortality, which may be reduced by avoiding crossing inhospitable ecological barriers. Despite flourishing avian migration research in recent decades, little is still known about inter-individual variability in migratory routes, as well as the carry-over effects of spatial and temporal features of migration on subsequent migration stages.

**Methods:**

We reconstructed post- and pre-breeding migration routes, barrier crossing behaviour and non-breeding movements of the largest sample (N = 85) analysed to date of individual barn swallows breeding in south-central Europe, which were tracked using light-level geolocators.

**Results:**

Most birds spent their non-breeding period in the Congo basin in a single stationary area, but a small fraction of itinerant individuals reaching South Africa was also observed. Birds generally followed a ‘clockwise loop migration pattern’, moving through the central Mediterranean and the Sahara Desert during post-breeding (north to south) migration yet switching to a more western route, along the Atlantic coast of Africa, Iberia and western Mediterranean during the pre-breeding (south to north) migration. Southward migration was straighter and less variable, while northward migration was significantly faster despite the broader detour along the Atlantic coast and Iberia. These patterns showed limited sex-related variability. The timing of different circannual events was tightly linked with previous migration stages, considerably affecting migration route and speed of subsequent movements. Indeed, individuals departing late from Africa performed straighter and faster pre-breeding migrations, partly compensating for the initial departure delays, but likely at the cost of performing riskier movements across ecological barriers.

**Conclusions:**

Different spatiotemporal migration strategies during post- and pre-breeding migration suggest that conditions *en route* may differ seasonally and allow for more efficient travelling along different migration corridors in either season. While highlighting patterns of inter-individual variability, our results support increasing evidence for widespread loop migration patterns among Afro-Palearctic avian migrants. Also, they suggest that carry-over effects acting across different phases of the annual cycle of migratory species can have major impacts on evolutionary processes.

## Introduction

Migratory species display a remarkable variability of directional movements when commuting between areas occupied during different phases of their life cycle. The spatio-temporal organization of migratory routes is expected to result from an optimization process that maximizes survival prospects and, ultimately, fitness [1]. Physiological requirements, environmental conditions and geographical features may constrain the choice of specific migratory routes, implying that actual routes may reflect trade-offs between the shortest possible paths and those which minimize the risk of migration-related mortality.

Migratory routes of long-distance migratory birds are largely shaped by so-called ecological barriers, i.e. vast areas of inhospitable or highly unsuitable habitats. Among terrestrial bird migrants, significant barriers are represented by stretches of open sea or deserts, which offer no or very limited, risky opportunities for landing and stopover during migratory journeys [2–6]. Mortality rates of migratory terrestrial bird species are generally higher during migration [7], and GPS tracking of individual terrestrial bird migrants has revealed that most mortality events across the annual cycle indeed occur while crossing these barriers [8].

Different bird species are known to adopt different strategies of barrier crossing depending on their physical abilities, the geographical features of the barrier, and the season when migration occurs. These barriers may indeed determine an uneven distribution of suitable stopover sites and food resources between seasons, as well as of favourable winds, updrafts, and location of suitable migration ‘bottlenecks’ (i.e., areas where birds congregate in large number to avoid barrier crossing). Such seasonal differences could foster so-called ‘loop migration’ patterns, whereby post- and pre-breeding routes (that is, Europe to Africa and Africa to Europe) are spatially separated by broad longitudinal gaps, largely determined by the variable spatiotemporal location of ecological barriers (e.g., [9–11]. Alternatively, loop migration may simply reflect the legacy of historical colonization routes [12].

In the Afro-Palearctic migration system, populations from different geographical regions may use different flyways to move between European breeding and sub-Saharan non-breeding areas [13, 14]. Birds from central-western Europe migrate south mainly through Iberia and the Atlantic coast of Africa (western flyway), or the Italian peninsula and the central Sahara (central flyway), while eastern European populations move across the Balkans, the Eastern Mediterranean and the Middle East (eastern flyway). Some populations select different pathways during pre- and post-breeding movements, thus performing loop migrations, while others follow similar routes in both autumn and spring. In the first case, the occurrence, extent and orientation (i.e., clockwise or anticlockwise) of loop migration vary considerably among populations [12, 15–21]. Inter-individual variation among birds from the same population may be similarly broad [22, 23]. For instance, individual barn swallows (*Hirundo rustica*) breeding in central Europe may combine different orientation routes in a single migration episode (in a clockwise or anticlockwise loop migration pattern), while others may follow similar routes during both post- and pre-breeding migration [22]. Such inter-individual variation in migration routes and behaviour may be shaped by carry-over effects, emerging whenever events at a given stage of the life cycle affect subsequent ones [24, 25]. Carry-over effects in migratory birds may involve breeding season effects on subsequent timing and organization of migration [26, 27] or the non-breeding period effects on subsequent migratory and breeding performance [28–30]. For instance, individuals departing late for migration or those forced to stopover because of adverse weather may alter their subsequent migratory journey (e.g. by migrating faster) to avoid immediate negative fitness consequences (due to e.g. increasing chances of experiencing adverse ecological conditions, reduced opportunities for breeding or establishing territories, etc.) (e.g. [31, 32]). Further, males and females may differ in migratory schedules or migratory tactics due to sex specific selective pressures [33]. Such differences, either due to natural or sexual selection, often result in earlier arrival of males relative to females to the breeding grounds (i.e., protandry, [34, 35], which may originate at different previous stages of the migration cycle, i.e., at departure for spring migration ([14, 36]).

Upon reaching their non-breeding areas, migrant terrestrial birds may perform a variety of additional movements. While some species are known to establish non-breeding territories and move very little before their pre-breeding, Africa to Europe migration [37], others may move considerable distances among different non-breeding locations, resulting in more itinerant behaviours, associated with the exploitation of ephemeral food resources and their spatio-temporal variation. This is the case, for example, of migratory raptor species that spend the non-breeding season in Sahelian drylands, then gradually move south/south-west during the non-breeding period to track shifting prey availability (e.g. [38]. Similarly, Nearctic-Neotropical migratory songbirds may move considerable distances during the non-breeding period to counteract the deterioration of local resource availability [39].

Here we reconstructed post-breeding (Europe to Africa) and pre-breeding (Africa to Europe) migration routes and non-breeding movements of the largest to date sample (N = 85) of individual barn swallows, aerial insectivores and diurnal migrants, breeding in South-central Europe (Po Plain and the pre-Alpine region, Italy and Switzerland) and equipped with miniaturized light-level geolocators (GLS, see [40]. Barn swallows from the target population migrate mostly along the central flyway in autumn and along the western flyway in spring [41–43] and largely spend the boreal winter in the Congo basin, with some individuals reaching South Africa [40–42, 44]. Part of the dataset used here was analysed in previous studies tackling variation in the location of the non-breeding areas, the migration timing and their carry-over effects on fitness (e.g. [40, 45, 46]. In those earlier studies, however, no accurate reconstruction of migration routes was presented because of technical limitations at the time. Our entirely novel analyses aim at: i) providing a detailed description of the migratory routes, barrier crossing behaviour, non-breeding movements, and sex differences in route characteristics, while accounting for variation among years and among individuals breeding in distinct study areas (detailed in Liechti et al. [40]; ii) assessing the carry-over effects of spatial and temporal features of migration on subsequent migration decisions (e.g. assessing how late departure affects migration route characteristics and migration speed, or how route characteristics affect arrival date; iii) assessing differences in migration features between post- and pre-breeding migration. As a priori predictions, in accordance with the time-minimization hypothesis, we expected birds to migrate at a faster pace during pre- than post-breeding migration (e.g., [47]). Moreover, individuals departing late may adopt straighter routes and travel at higher speeds during both spring and autumn [32]. Although in previous studies of these populations no major sex differences in timing of the life cycle events emerged [40], we explored here whether route characteristics of males and females differed, possibly reflecting differences in morphology and aerodynamic costs of flight between the sexes [48].

## Methods

### Study area and field methods

Data were collected over four breeding seasons (2010–2013) in three study areas, the northernmost one being in southern Switzerland, hereafter “N” area (centred at Magadino, 46°09′N, 8°55′E, 211 m a.s.l.) and the other two in northern Italy, hereafter “SW” (Piedmont region, 45°33′N, 8°44′E, 160 m a.s.l.) and “SE” areas (Lombardy region, 45°19′N, 9°40′E, 60 m a.s.l.), respectively. Capture, ringing and GLS deployment were performed in accordance with the Swiss and Italian regulations concerning scientific investigations on bird species in the wild and approved by the Office Fédéral de l’Environnement (OFEV, Division Espèces, Ecosystèmes, Paysages; Switzerland, permit n. F044-0799), by the Provincia di Novara (auth. n. 905 issued on March 21, 2011), and by Regione Lombardia (auth. n. 329 issued on January 21, 2009, and n. 2141 issued on March 9, 2011). Details of study areas, field procedures, and GLS deployed are reported in Scandolara et al. [49] and Liechti et al. [40]. Breeding barn swallows were captured using mist-nets, ringed, and their standard biometrics recorded. We deployed SOI-GDL2.10-SOI-GDL2.11 tags (Swiss Ornithological Institute, CH) using an elastic silicone-rubber leg loop harness (mean weight = 0.73 g, ranging between 0.57 and 0.87 g including harness). The relative tag load was on average 3.9% of average body mass at deployment (2.7–5.1% minimum–maximum). GLS were deployed during the breeding seasons in 2010, 2011, and 2012 and retrieved from returning individuals during the subsequent season. Return rates of GLS-tagged birds varied between 0.08 and 0.40, depending on year and sex (details in [49]. For simplicity of notation, when mentioning different years, we will systematically refer to the year of GLS deployment. We considered a single return migration episode for each individual. Details on the number of individuals for which we reconstructed post- and pre-breeding migration routes (by study area and year) are reported in Table 1.

**Table 1:** Number of individuals with complete migration routes according to year (group of columns) and population of origin (rows)

Study area	Year 2010			Year 2011			Year 2012			Total
	Post-br	Pre-br	Both	Post-br	Pre-br	Both	Post-br	Pre-br	Both	
SE	3	3	(3)	11	10	(9)	–	–	–	15 (12)
SW	25	15	(15)	4	5	(4)	2	2	(2)	30 (21)
N	30	18	(18)	8	7	(7)	–	–	–	38 (25)
Total	58	36	(36)	23	22	(20)	2	2	(2)	85 (58)

The number of individuals with complete routes during both post- and pre-breeding migrations is reported in brackets. Note that totals per study area do not necessarily correspond to sums of post- and pre-breeding migrations because one GLS can provide complete data for the post-breeding migration route only and another for the pre-breeding route only

### Light-level data analysis

GLS are tracking devices that record daylight to estimate location [6]. They are currently the lightest available tracking devices and the only ones suitable for tracking small birds. A GLS records also the time of light measures thanks to an internal clock. Circadian variability in light-level profiles were inspected to infer departure/arrival dates from/to the breeding site as detailed in Liechti et al. [40]. To identify migratory routes, we relied on the R package *FLightR* (ver. 0.4.6, [50], which, basically, tries to infer position from the timing of twilight events extracted from light level profiles. Thus, GLS data allow estimating two positions per day at maximum (at sunrise, and sunset). In additiom, *FLightR* incorporates the uncertainty of the position estimates by simulating the migration path through an uncorrelated random walk movement model and adjusting the trajectories at each twilight event using a Particle Filter algorithm (for a detailed explanation of the procedure see [51]. As *FLightR* requires not only the timing of twilight events, but also a clear light-level profile around sunsets and sunrises, we pre-filtered twilight events as described in Pancerasa et al. [52] using the *TwGeos* R package (ver. 0.1.2, [53]. Upon visual inspection, twilights showing a neat light-level profile (with no obvious disturbance from shadings and/or bird roosting behaviour) were considered for route reconstruction. We generated a spatial grid that allowed a bird to fly overwater a maximum of 300 km and to rest at a maximum of 50 km from the shoreline. We then estimated the likelihood of bird positions on the grid at each twilight event by providing to FLighR the light data processed by TwGeos, the results of the calibration procedure, the specifications of the spatial extent and the location of application and removal of the GLS from the individual. We estimated the migratory route of each individual using the particle filter procedure (i.e., generating 1,000,000 simulated routes per bird based on the above-mentioned likelihoods and averaging the geographical positions at each twilight event, binding the spatial simulation of complete tracks to end within 25 km of the recapture site). We used default parameters (E. Rhakimberdiev, pers. com.), relying on pre-deployment light-level calibration data. To identify the periods when a bird pauses migration, the so-called stationary periods, we adopted the procedure that was specifically developed for barn swallow GLS data by Liechti et al. [40]. Such procedure relies on the assumption that, if a bird is stationary, twilights should follow a smooth natural seasonal trend in time, whereas if a bird migrates to a different site, this short-term temporal trend should somehow show some discontinuities (see [40] for details and its associated R script). Stationary periods were thus identified based on variations in the time of twilight events because this procedure has proven to be more robust than one based on estimated positions. Compared to Liechti et al. [40], here we did not weigh data according to twilight quality, as we already pre-filtered data so as to select high-quality twilight events only, as described above. The geographic location of stationary periods was computed as the centre of density (mode, with 90% quantiles) of both daily longitude and latitude estimates obtained from FlighR route reconstructions during those periods. Consecutive stationary periods whose modal distances were < 200 km (based on *undefined* route reconstructions) were merged and the location of their centre of density re-calculated on the merged data. Likewise, stationary periods shorter than 14 days (migration stopovers, n = 22 out of 111 non-breeding stationary periods) were not considered due to the poor accuracy of GLS data [40]. The southern boundary of the Sahara Desert (identified as the geographic boundary with the rainfall threshold of 200 mm y^−1^) is considered to be located at ca. 17° N in the central-western portion of the desert [54]. We thus considered 17° N as the southern margin of the Sahara Desert. All non-breeding stationary periods were indeed located south of 17° N (the northernmost stationary period was at 15.6° N, in the Inner Niger Delta region, Mali). Latitudes and longitudes of the longest stationary periods for each individual in the non-breeding residence areas, as obtained in this study using *FLightR*, were strongly positively correlated with those reported in [40] (latitude: Pearson’s *r* = 0.98; longitude: *r* = 0.99) using a different procedure based on the *GeoLight* R package [55]. Compared to Liechti et al. [40], the mean estimated positions had a similar longitude (paired t-test, t_86_ = 1.48, *p* = 0.15), while present latitudinal estimates obtained with *FLightR* were on average 1.56° (ca. 174 km) more southern than previous estimates obtained with GeoLight (t_86_ = 8.9, *p* < 0.001).

Key to performing our analyses were the following variables retrieved from individual tracks:

- Departure from (departure date, “DD”) and arrival to (arrival date, “AD”) the target locations of the migratory journey, i.e., the breeding colony and the first/last stationary period south of the Sahara (see below). Day 1 is January 1st. Departure/arrival to breeding colony assessed here as in Liechti et al. [40],
- Length of migration path (“LMP”, in km): variable calculated separately for post- and pre-breeding migration as the sum of all the step-by-step distances covered by an individual along its migration path reconstructed using FlightR. The initial and final coordinates of the migration path were respectively set as follows: for post-breeding migration, the breeding colony and the first stationary period south of Sahara; for pre-breeding migration, the last stationary period south of Sahara and the breeding colony;
- Path straightness (“PS”, %): ratio between LMP and the length of the great-circle distance [56] between the breeding colony and the first/last stationary period of a migration journey (post- or pre-breeding),
- Duration of migration (“DM:, days): difference in days between AD and DD;
- Migration speed (“MS”, km day^−1^): ratio between LMP and DM; It is worth emphasizing that, MS reflects the average number of kilometres travelled in one day, and should not be confused with "flight speed”, which is the speed during flight, a quantity that cannot be measured with GLS data.
- Coordinates (latitude and longitude) of the first (after post-breeding migration) or last (before pre-breeding migration) stationary period south of Sahara. Although most individuals had a single stationary period in their African non-breeding grounds (hence the location of first and last stationary periods coincided for most individuals), very few of them (see Results) had two distinct stationary periods; in such cases, the longest one was considered as the main stationary period for that individual. These few birds with two stationary periods were defined as ‘itinerant’, whereas the others were defined as ‘stationary’.

Abbreviations may be accompanied by different subscripts to identify specific events (e.g. MS_*pob*_ = post-breeding migration speed, AD_*sp*_ = arrival date to the first stationary period, PS_*prb*_ = path straightness during pre-breeding migration, AD_*c*_ = arrival date to the colony site, SP_*f*_ = first stationary period, SP_*l*_ = last stationary period).

### Spatial consistency of migration routes

To compute the similarity of two migration paths, we used the One-Way-Distance (“OWD”) metric ([57], see also [58]) considering only the sections of the tracks that were between 17° N and 45° N (i.e. just to the south of the southernmost breeding area) to avoid the effect of different migratory routes lengths on OWD calculations. As the OWD (see below) is sensitive to the number of locations composing each track, we equalized the sampling points of the post-breeding and pre-breeding routes of each track by selecting 20 equally distanced locations on it. We then computed OWD between track T_A_ and track T_B_ (OWD_AB_) as follows: i) we calculated the distance between the two tracks *d*_AB_ as the sum of the great circle distances between each of the 20 selected positions of T_A_ and their corresponding positions in T_B_, and divided the result by the total length of T_A_; the corresponding position of a location on track A is the location on track B (within its 20 locations) having the minimum great circle distance; ii) we calculated *d*_BA_ using the same procedure as in the previous point, but swapping T_A_ and T_B_; iii) we computed OWD_AB_ as the mean value of *d*_AB_ and *d*_BA_.

### Statistical analyses

All statistical analyses were run using R 3.6.2 (R Core Team 2020). Analyses of factors affecting migration features were performed by linear models fitted separately to post- and pre-breeding migration data. The general purpose of these models was to assess whether migration features varied among sexes (2-level factor) and whether they were affected by carry-over effects, while accounting for differences among years (2-level factor) and study areas (3-level factor) [40]. To investigate the carry-over effects of specific migration features on subsequent individual spatial and temporal migration patterns, we added to these models specific spatial and temporal covariates as reported in Table 2. Since we fitted linear models for arrival date and migration speed, we did not fit models for duration of migration because they would largely be redundant.

**Table 2:** List of dependent variables and of temporal and spatial covariates included in linear models of factors explaining variation in post- and pre-breeding migration features

Dependent variable	Temporal covariates	Spatial covariates
*Post-breeding migration*		
Length of migration path (LMP_*pob*_)	DD_*c*_	–
Path straightness (PS_*pob*_)	DD_*c*_	–
Migration speed (MS_*pob*_)	DD_*c*_	PS_*pob*_
Latitude of the first stationary period (Lat-SP_*f*_)	DD_*c*_, MS_*pob*_	PS_*pob*_
Longitude of the first stationary period (Lon-SP_*f*_)	DD_*c*_, MS_*pob*_	PS_*pob*_
Arrival date to the first stationary period (AD_*sp*_)	DD_*c*_, MS_*pob*_	PS_*pob*_, Lat-SP_*f*_, Lon-SP_*f*_
*Pre-breeding migration*		
Departure date from the last stationary period (DD_*sp*_)	AD_*sp*_	Lat-SP_*l*_, Lon-SP_*l*_
Length of migration path (LMP_*prb*_)	DD_*sp*_	Lat-SP_*l*_, Lon-SP_*l*_
Path straightness (PS_*prb*_)	DD_*sp*_	Lat-SP_*l*_, Lon-SP_*l*_
Migration speed (MS_*prb*_)	DD_*sp*_	PS_*prb*_, Lat-SP_*l*_, Lon-SP_*l*_
Arrival date to the breeding colony (AD_*c*_)	DD_*sp*_, MS_*prb*_	PS_*prb*_, Lat-SP_*l*_, Lon-SP_*l*_

Spatial and temporal covariates were included to assess the carry-over effects of previous migration events/route features on specific migration features. *DDc*  Departure date from the colony site

Once we found that one variable was successfully predicted by others, when elaborating more complex models that account also for that explained variable, we did not incorporate it *as is* into the novel model. Rather, we incorporated only the residuals to decouple the effect of that explained variable from that of the others that are collinear with it.

If the explained variable was significantly predicted by a fixed factor (e.g. in case of variables differing among years), we group-centred that explained variable (subtracting from each value of a group the mean value for that group). When feasible, group-centring is preferable to using residuals, because least-square means from linear models do not correspond to group means for unbalanced designs [59]. The use of residuals or group-centred values effectively reduced collinearity of predictors (variance inflation factor always < 1.8). Significance was assessed by permutation tests (10,000 permutations) using the *aovperm* procedure of the *permuco* R package [60]. We then investigated whether the probability of an individual being itinerant was affected by sex, year and study area, as well as by latitude and longitude of SP_*f*_, and by AD_*sp*_, using a binomial generalized linear model (GLM).

Within-individual differences between post- and pre-breeding migration features were tested using linear mixed models (LMMs). Fixed effects were migration period (2-level factor, post- or pre-breeding), sex, year and study area, as well as the two-way interactions between migration period and all the other predictors. Individual identity was included as a random factor, while migration period was considered as a random slope within individual. The random structure henceforth ensures within-individuals pairwise comparisons of migration features. Models were fitted using the *lme* procedure of the *nlme* R package [61]. Significance was assessed by a permutation test as follows. First, we randomly shuffled post- and pre-breeding migration parameters within individuals only. Second, we randomly re-assigned both the (possibly rearranged) migration parameter values of one individual to a different individual, thus preserving the intrinsic correlated nature of the dataset. At each step, the value of the likelihood ratio tests (LRT) of the effect of each predictor was noted and significance was assessed based on the rank of the LRT-value of the model fitted to the observed data compared to the distribution of those obtained from the randomization procedure (10,000 permutations).

Whenever significant differences among populations in migration features emerged, we performed *post-hoc* tests with the Tukey method using the *multcomp* R package [62].

To assess the spatial consistency of migration routes of the same individuals between seasons, we compared the OWDs between the post- and pre-breeding route of the same individual with the OWDs between the post-breeding migration of one individual and the pre-breeding one of another. Moreover, to assess the difference in the spatial consistency of migration routes between seasons, we compared the OWDs between pre-breeding migration routes of all individuals with the OWDs between post-breeding routes.

Tests of OWD differences were performed by a permutation procedure (10,000 permutations) using the R *permuco* package (ver. 1.1.0) [60].

Overall, we reconstructed the migration routes of 85 individuals, 58 of which had complete post- and pre-breeding migration routes (Table 1). We excluded the two individuals from year 2012 from all statistical comparisons to avoid including an additional level to the year factor in linear models. Similarly, we excluded from all statistical comparisons four individuals that had clearly distinct migration patterns and migrated to non-breeding areas located south of 10° S in southern Africa.

## Results

### General description of migration routes and barrier crossing

**Fig. 1:**
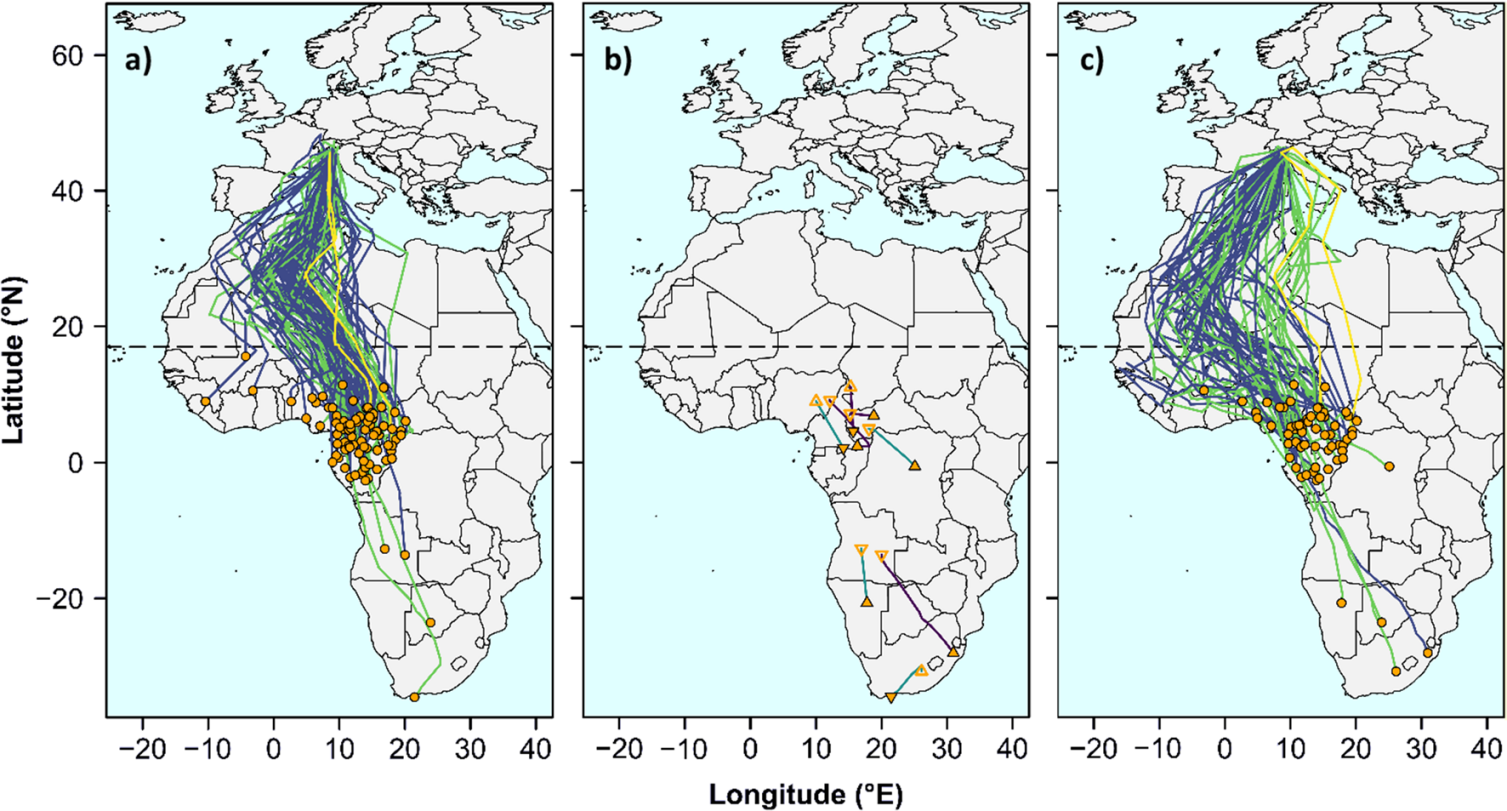
Migration routes of barn swallows breeding in south-central Europe (northern Italy and southern Switzerland) in 2010–2012. **a** Post-breeding (Europe–Africa) autumn migration routes (n = 73); **b** regional-scale movements between multiple stationary periods in Africa during boreal winter of itinerant individuals (n = 8); **c** pre-breeding (Africa to Europe) spring migration routes (n = 60). Trajectory colours represent different years (darker: 2010; lighter 2012). Dots indicate position of the first (post-breeding migration) or the last (pre-breeding migration) stationary period north of the 45° N (where the SE, SW and N populations are located) or south of 17° N (broken horizontal line, southern margin of the Sahara Desert; see Methods). Triangles in panel b represent the position of the main non-breeding stationary period (filled triangle) and of the secondary stationary period (open triangle) of itinerant individuals; downward pointing triangles: first non-breeding stationary period south of 17° N; upward pointing triangles: last non-breeding stationary period south of 17° N

The migration routes we reconstructed are shown in Fig. 1, while summary statistics of spatial and temporal migration features (including sample sizes by year) are reported in Table 3. The length of migration paths (LMP) of most individuals varied between ca. 5500–6000 km for both post- and pre-breeding migration, but some birds moved over 9,000 km (Table 3). The longest reconstructed migration path was 12,899 km (pre-breeding migration of a bird with a South African non-breeding area, Fig. 1).

**Table 3:** Summary statistics (mean ± SE) of migration timing, route features and movement patterns (see Methods) for different groups of individuals

	Individuals included in statistical analyses (years 2010–2011, sample size in brackets)	Individuals with non-breeding areas south of 10° S, (n = 4)	Individuals from year 2012 (n = 2)
*Post-breeding migration*			
Departure date (DD_*c*_)	250.77 ± 0.75 (81)	250.25 ± 0.63	253.5 ± 0.5
Length of migration path (LMP_*pob*_) (km)	5557.78 ± 83.81 (81)	8712.85 ± 794.99	5191.83 ± 629.93
Path straightness (PS_*pob*_)	83.88 ± 0.78 (81)	86.51 ± 1.71	91.16 ± 4.11
Migration speed (MS_*pob*_) (km day^−1^)	207.65 ± 7.59 (81)	187.92 ± 10.43	248.35 ± 72.92
Duration of migration (DM_*pob*_) (d)	29.52 ± 1.09 (81)	46.42 ± 3.53	23.69 ± 9.49
Latitude of the first SP	4.48 ± 0.40 (83)	− 21.11 ± 5.11	3.65 ± 3.05
Longitude of the first SP	12.27 ± 0.56 (83)	20.58 ± 1.46	16.33 ± 1.70
Arrival date to the first SP (AD_*sp*_)	280.09 ± 0.99 (81)	296.50 ± 3.75	277.00 ± 9.00
*Sub-Saharan Africa itinerancy*			
Latitude of the main non-breeding SP	4.33 ± 0.40 (83)	− 26.73 ± 3.012	3.65 ± 3.05
Longitude of the main non-breeding SP	12.45 ± 0.58 (83)	23.53 ± 2.78	16.33 ± 1.70
Duration of stay	163.51 ± 1.38 (56)	141.53 ± 7.05	159.52 ± 9.00
Path length (km)^a^	990.65 ± 165.42 (8)	–	–
Movement speed (km day^−1^)^a^	6.68 ± 1.29 (8)	–	–
Path straightness^a^	92.72 ± 4.18 (8)	–	–
*Pre-breeding migration*			
Latitude of the last stationary period	4.48 ± 0.41 (83)	− 25.79 ± 2.26	3.65 ± 3.05
Longitude of the last stationary period	12.31 ± 0.57 (83)	24.69 ± 2.73	16.33 ± 1.70
Departure date from the last SP (DD_*sp*_)	80.10 ± 1.48 (58)	72.50 ± 6.59	70 ± 0
Length of migration path (LMP_*prb*_) (km)	6154.64 ± 131.37 (58)	9358.12 ± 591.14	5184.49 ± 72.92
Path straightness (PS_*prb*_)	76.83 ± 1.48 (58)	87.27 ± 4.58	90.80 ± 0.16
Migration speed (MS_*prb*_)	296.47 ± 15.53 (57)	239.89 ± 33.02	243.57 ± 19.02
Duration of migration (DM_*prb*_) (d)	23.69 ± 1.26 (57)	40.80 ± 4.94	21.28 ± 0.00
Arrival date (AD_*c*_)	104.84 ± 1.51 (57)	114.00 ± 5.70	92 ± 0

a: includes all itinerant individuals, i.e., also those with non-breeding areas south of 10° S

Round-trip migration routes generally showed a clockwise loop migration pattern, with individuals mostly following the central flyway during post-breeding migration and then shifting to the western flyway during pre-breeding migration (Fig. 1, Table 3). During post-breeding migration, most individuals moved southwards through Corsica and Sardinia, entered Africa through Tunisia and crossed the Sahara Desert with a route that shifted initially westwards and then bent eastwards when approaching the southern margin of the desert. A minority of individuals followed the Tyrrhenian coast, crossing the Mediterranean at the Balearic Islands or even at Gibraltar. No bird in our sample migrated south along the Italian peninsula (Fig. 1a).

Four individuals spent the boreal winter at exceptionally southern latitudes (south of 10° S) (Table 3). Eight individuals (including three of the four mentioned above) behaved as itinerants upon reaching sub-Saharan Africa (Fig. 1b).

Pre-breeding migration routes showed larger westward detours compared to post-breeding ones, with individuals crossing the Mediterranean Sea almost uniformly between Gibraltar and Malta while some moved eastwards through Sicily and the Italian peninsula (Fig. 1c).

### Post-breeding migration features

Post-breeding migration features did not significantly differ between males and females (Table 4). There was a positive relation between migration speed and the southern latitude of the non-breeding area, and individuals departing later travelled at a faster pace than those departing earlier. Individuals departing later from the breeding colonies tended to reach their first stationary area later, but the slope of this effect (0.41 ± 0.06 SE) was significantly lower than 1 (t_71_ = − 9.22, *P* < 0.001), implying that, on average, each day of delay in leaving the breeding colonies translated into a delay of less than half a day in reaching their non-breeding areas. Individuals arriving earlier to their non-breeding areas followed straighter routes, travelled at a faster pace and settled at more northern non-breeding areas (Table 4).

**Table 4:** Linear models of post-breeding migration features

Predictor	F	d.f	P	Least-square means (SE) / slopes (SE)		
*Departure date (DD *_c_*) (n* = *81)*						
Sex	3.37	1,76	0.064			
Year	**10.2**	**1,76**	**0.002**	**2010: 252.10 (1.05)**	**2011: 246.32 (1.38)**	
Study area	0.03	2,76	0.963			
*Length of migration path (LMP*_pob_*) (n* = *81)*						
Sex	0.21	1,75	0.650			
Year	**6.53**	**1,75**	**0.015**	**2010: 5406 (120)**	**2011: 5934 (157)**	
Study area	1.04	2,75	0.369			
c-DD	1.48	1,75	0.231			
*Path straightness (PS*_pob_*) (n* = *81)*						
Sex	0.32	1,75	0.563			
Year	0.29	1,75	0.755			
Study area	2.46	2,75	0.121			
c-DD	0.06	1,75	0.803			
*Migration speed (MS*_pob_*) (n* = *81)*						
Sex	0.03	1,74	0.858			
Year	**15.95**	**1,74**	**< 0.001**	**2010: 239.30 (10.34)**	**2011: 167.68 (13.61)**	
Study area	**5.06**	**2,74**	**0.008**	**SE: 247.36 (17.47)**^**a**^	**SW: 184.79 (13.39)**^**b**^	**N: 178.31 (11.32)**^**b**^
c-DD	**6.05**	**1,74**	**0.017**	**2.75 (1.12)**		
PS	0.71	1,74	0.398			
*Latitude of the first stationary period (Lat-SP*_f_*) (n* = *81)*						
Sex	0.05	1,73	0.823			
Year	**4.25**	**1,73**	**0.043**	**2010: 4.77 (0.55)**	**2011: 2.80 (0.73)**	
Study area	1.94	2,73	0.154			
c-DD	2.49	1,73	0.116			
PS	0.53	1,73	0.463			
r-MS	**4.36**	**1,73**	**0.043**	**− 0.01 (0.01)**		
*Longitude of the first stationary period (Lon-SP*_f_*) (n* = *81)*						
Sex	0.30	1,73	0.590			
Year	0.71	1,73	0.409			
Study area	0.56	2,73	0.573			
c-DD	0.09	1,73	0.766			
PS	0.11	1,73	0.743			
r-MS	1.11	1,73	0.296			
*Arrival date to the first stationary period (AD*_f_*) (n* = *74)*						
Sex	1.04	1,71	0.313			
Year	**36.87**	**1,71**	**< 0.001**	**2010: 276.64 (0.58)**	**2011: 282.84 (0.77)**	
Study area	**32.71**	**2,71**	**< 0.001**	**SE: 273.63 (0.99)**^**a**^	**SW: 283.65 (0.63)**^**b**^	**N: 281.93 (0.76)**^**b**^
c-DD	**40.91**	**1,71**	**< 0.001**	**0.41 (0.06)**		
PS	**28.74**	**1,71**	**< 0.001**	**− 0.30 (0.06)**		
r-MS	**290.94**	**1,71**	**< 0.001**	**− 0.11 (0.01)**		
r-Lat-SP	**18.94**	**1,71**	**< 0.001**	**− 0.58 (0.13)**		
Lon-SP	0.19	1,71	0.658			

**Fig. 2:**
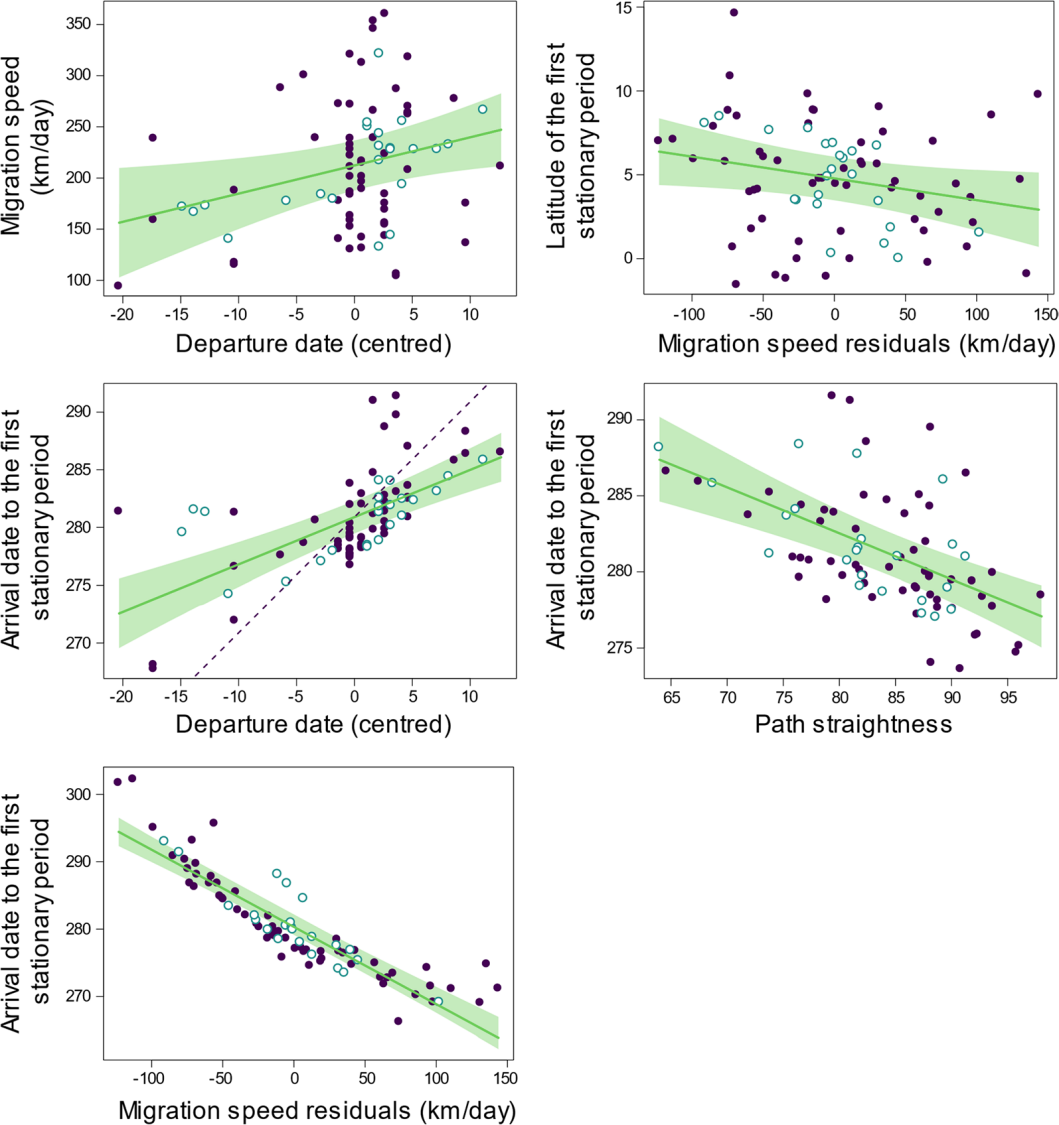
Effects of temporal predictors on barn swallow post-breeding migration route characteristics. Only covariates that were statistically significant in linear models reported in Table 4 are shown (in the same order as they appear in Table 4). Fitted lines (with 95% confidence bands) are derived from the analyses displayed in Table 4; broken lines represent a slope = 1 for comparison (when relevant; see Results). Filled dots: 2010; empty dots: 2011. See methods for how centred variables and residuals were calculated

Different superscript letters indicate significant (*P* < 0.05) pairwise differences of least-square means at Tukey *post-hoc* tests. Statistically significant predictors are highlighted in boldface. c-: centred variable; r-: residuals. the effects of significant temporal variables on pre-breeding migration features are shown in Fig. 2. See methods for how centred variables and residuals were calculated

Post-breeding migration features also showed significant differences among study areas and years. Birds left their breeding colony earlier (by ca. 6 days), migrated farther (by ca. 500 km), but on average more slowly (by ca, 71.7 km/day). and reached their non-breeding area later (by ca. 6 days) in 2011 compared to 2010 (Table 4). Moreover, birds from the SE study area migrated faster and reached their non-breeding area earlier than those from other study areas (Table 4).

### Sub-Saharan Africa itinerancy

Three of the eight individuals classified as itinerant had both stationary periods south of 10° S. The others had both stationary periods north of 10° S (Fig. 1b). For five itinerant individuals, the main stationary period coincided with the first one south of the Sahara Desert, while for the other three it coincided with the last one. In most cases, the longest stationary period was the southernmost one (Fig. 1b). None of the variables we entered in the analysis predicted itinerant behaviour (Table 5). The speed of movement of itinerant birds was much slower (mean ± SE = 6.68 ± 1.29 km day^−1^) than post- and pre-breeding migration speed of the same individuals (respectively 204.78 ± 14.33 km day ^−1^ and 260.19 ± 25.86 km day ^−1^). The direction of these movements was also highly variable among individuals (Fig. 1b).

**Table 5:** Binomial generalized linear model of the factors affecting the probability of an individual being itinerant in sub-Saharan Africa

Predictors	χ^2^	d.f	*P*
Sex	1.92	1	0.17
Year	0.14	1	0.71
Study area	0.82	2	0.66
c-Lat-SP	0.22	1	0.64
Lon-SP	2.87	1	0.09
c-AD	0.06	1	0.81

c-: centred variable; SP: stationary period; AD: Arrival date. See methods for how centred variables were calculated

### Pre-breeding migration features

Similarly to the analysis of post-breeding migration, we found no significant differences between males and females in pre-breeding spatial and temporal migration features (Table 6). There was a longitudinal cline in departure date from the non-breeding areas, individuals with more eastern non-breeding areas departing earlier (Table 6). Individuals that had reached their non-breeding areas later in boreal autumn also departed later on pre-breeding migration (Table 6), and the slope of this relationship did not differ significantly from 1 (t_48_ = − 1.59, *P* = 0.12). Hence, individuals tend to spend an equal amount of time in sub-Saharan Africa irrespective of their arrival date to their non-breeding areas.

**Table 6:** Linear models of pre-breeding migration features

Predictor	F	d.f	*P*	Least-square means (SE) / slopes (SE)		
*Latitude of the last stationary period (Lat-SP*_l_*) (n* = *81)*						
Sex	0.00	1,75	0.981			
Year	**4.00**	**1,75**	**0.047**	**2010: 4.78 (0.59)**	**2011: 2.74 (0.78)**	
Study area	1.58	2,75	0.219			
c-AD	0.29	1,75	0.593			
*Longitude of the last stationary period (Lon-SP*_l_*) (n* = *81)*						
Sex	0.03	1,75	0.861			
Year	0.73	1,75	0.399			
Study area	0.58	2,75	0.558			
c-AD	1.31	1,75	0.259			
*Departure date from the last stationary period (DD*_sp_*) (n* = *56)*						
Sex	0.90	1,48	0.349			
Year	**6.90**	**1,48**	**0.012**	**2010: 76.74 (1.86)**	**2011: 84.52 (2.21)**	
Study area	0.91	2,48	0.414			
c-Lat-SP	1.31	1,48	0.267			
Lon-SP	**7.74**	**1,48**	**0.007**	**− 0.84 (0.30)**		
c-AD	**18.62**	**1,48**	** < 0.001**	**0.73 (0.17)**		
*Length of migration path (LMP*_prb_*) (n* = *56)*						
Sex	0.23	1,48	0.637			
Year	0.60	1,48	0.447			
Study area	2.41	2,48	0.101			
c-Lat-SP	**13.02**	**1,48**	**0.001**	**− 146.74 (40.67)**		
Lon-SP	0.30	1,48	0.581			
r-DD	**7.66**	**1,48**	**0.007**	**− 37.80 (13.66)**		
*Path straightness (PS*_prb_*) (n* = *56)*						
Sex	0.16	1,48	0.686			
Year	1.32	1,48	0.263			
Study area	1.80	2,48	0.173			
c-Lat-SP	0.19	1,48	0.669			
Lon-SP	0.60	1,48	0.448			
**r-DD**	**7.45**	**1,48**	**0.008**	**0.43 (0.16)**		
*Migration speed (MS*_prb_*) (n* = *55)*						
Sex	0.95	1,46	0.339			
Year	1.82	1,46	0.183			
**Study area**	**4.13**	**2,46**	**0.019**	**SE: 348.66 (28.88)**^**a**^	**SW: 282.98 (22.62)**^**ab**^	**N: 243.73 (19.86)**^**b**^
c-Lat-SP	0.63	1,46	0.428			
Lon-SP	0.74	1,46	0.395			
**r-DD**	**13.26**	**1,46**	**< 0.001**	**5.12 (1.41)**		
r- PS	0.70	1,46	0.410			
*Arrival date (AD*_c_*) (n* = *54)*						
Sex	0.00	1,45	0.949			
**Year**	**23.57**	**1,45**	**< 0.001**	**2010: 100.20 (1.33)**	**2011: 110.70 (1.61)**	
Study area	2.55	2,45	0.090			
c-Lat-SP	3.26	1,45	0.079			
**Lon-SP**	**5.37**	**1,45**	**0.027**	**− 0.50 (0.21)**		
**r-DD**	**20.84**	**1,45**	**< 0.001**	**0.48 (0.11)**		
**r-PS**	**17.12**	**1,45**	**< 0.001**	**− 0.39 (0.09)**		
**r-MS**	**21.94**	**1,45**	**< 0.001**	**− 0.05 (0.01)**		

**Fig. 3:**
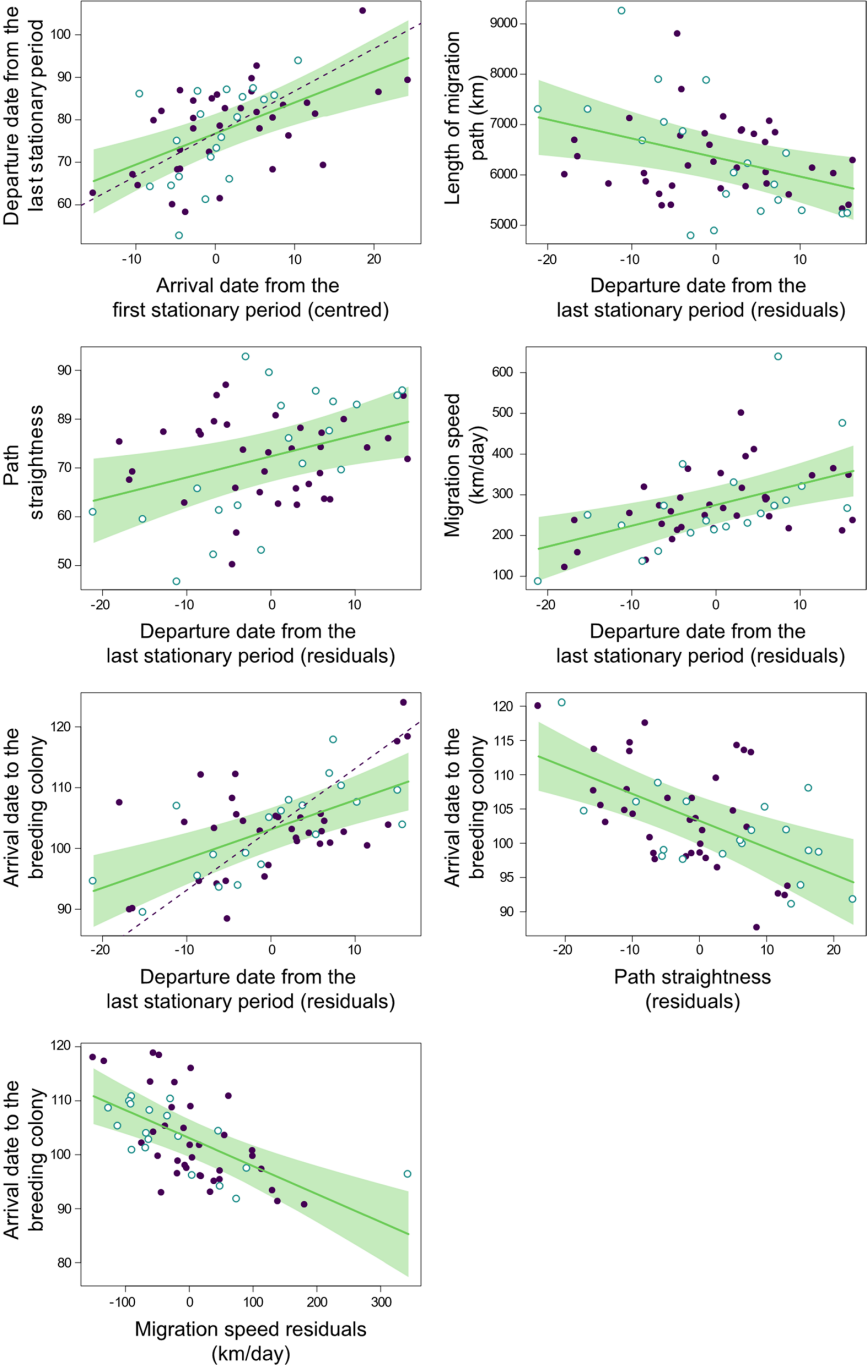
Effects of temporal predictors on barn swallow pre-breeding migration route characteristics. Only covariates that were statistically significant in the linear models displayed in Table 6 are shown (in the same order as they appear in Table 6). Fitted lines (with 95% confidence bands) are derived from the linear models displayed in Table 6; broken lines represent a slope = 1 for comparison (when relevant; see Results). Filled dots: 2010; empty dots: 2011. See Methods for how centred variables and residuals were calculated

Different superscript letters indicate significant (*P* < 0.05) pairwise differences of least-square means at Tukey *post-hoc* tests. Statistically significant predictors are highlighted in boldface. c-: centred variable; r-: residuals; the effects of significant temporal variables on pre-breeding migration features are shown in Fig. 3. See Methods for how centred variables and residuals were calculated

Late-departing individuals had shorter migration routes, even controlling for the effect of non-breeding latitude (Table 6). They also followed straighter migration routes and migrated at a faster pace (Table 6).

Birds arriving earlier at their colony site also showed an earlier departure from the non-breeding areas, but the slope of this association was significantly lower than 1 (t_45_ = − 46.63, *P* < 0.001), consistent with the hypothesized faster migration of late-departing individuals. In addition, earlier arriving birds had straighter migration paths and travelled at a faster pace. Finally, individuals with more eastern non-breeding areas arrived earlier to their breeding colony, consistent with their earlier departure (Table 6).

There were marked differences among years in both timing and location of non-breeding areas, birds departing later and from more southern non-breeding areas in 2011 compared to 2010, and arriving later to their breeding colony. Birds from different study areas were homogeneous for most migration features, the single exception being migration speed, which was faster for individuals of the SE than for those of the N population.

### Migratory behaviour of individuals with South African non-breeding areas

All the four individuals that migrated south of 10° S were males. Their migratory behaviour is summarized in Table 3. They migrated on average 3200 km and 16 days more than the others, and three of them were also itinerant. When in sub-Saharan Africa, they stayed on average 20 days less and departed 8 days earlier than other individuals. In spite of a relatively straighter migration path, they arrived at their breeding colonies ca. 10 days later than the other individuals. During both post- and pre-breeding migrations, they travelled at a slightly slower pace than the other individuals.

### Within-individual comparisons of post- and pre-breeding migration features and spatial consistency

Pre-breeding migration routes were significantly longer, by ca. 7% (i.e. ca. 450 km longer), than post-breeding routes (Fig. 1, Tables 3 and 6). Moreover, post-breeding migration routes were considerably straighter than pre-breeding ones (Fig. 1, Tables 3 and 7). Yet, during pre-breeding migration, birds travelled at a considerably faster pace compared to post-breeding migration (by 90 km day^−1^ faster, on average), resulting in a shorter duration of pre- (by ca. 7 d) compared to post-breeding migration (Table 7). Males and females had similar migration features in both seasons (the migration period × sex interactions were not significant in any of the analysed models; Table 7).

**Table 7:** Mixed models of intra-individual differences between post- and pre-breeding migration routes (n = 55 individuals)

Predictor	χ^2^	d.f	*P*	Least-square means (SE)		
*Length of migration path (LMP)*						
**Migration period (MP)**	**13.67**	**1**	**< 0.001**	**Post-br.: 5715 (117)**	**Pre-br.: 6152 (152)**	
Sex	0.04	1	0.850			
Year	3.12	1	0.091			
Study area	0.88	2	0.429			
MP × sex	0.67	1	0.407			
MP × year	1.16	1	0.283			
MP × study area	0.79	2	0.654			
*Path straightness (PS)*						
**Migration period (MP)**	**15.53**	**1**	**< 0.001**	**Post-br.: 82.92 (1.07)**	**Pre-br.: 78.09 (1.63)**	
Sex	0.07	1	0.791			
Year	0.11	1	0.756			
Study area	2.02	2	0.986			
MP × sex	1.08	1	0.303			
MP × year	2.90	1	0.092			
MP × study area	0.85	2	0.630			
*Migration speed (MS)*						
**Migration period (MP)**	**28.39**	**1**	**< 0.001**	**Post-br.: 201.99 (9.44)**	**Pre-br.: 293.78 (14.57)**	
Sex	0.18	1	0.678			
**Year**	**10.53**	**1**	**< 0.001**	**2010: 271.32 (11.26)**	**2011: 224.44 (13.15)**	
**Study area**	**17.8**	**2**	**< 0.001**	**SE: 300.40 (17.30)**^**a**^	**SW: 227.81 (14.28)**^**b**^	**N: 215.44 (12.25)**^**b**^
MP × sex	0.00	1	0.982			
MP × year	0.60	1	0.440			
MP × study area	0.16	2	0.938			
*Duration of migration (DM)*						
**Migration period (MP)**	**13.06**	**1**	**< 0.001**	**Post-br.: 31.23 (1.31)**	**Pre-br.: 24.01 (1.38)**	
Sex	0.36	1	0.565			
**Year**	**18.79**	**1**	**< 0.001**	**2010: 23.72 (1.16)**	**2011: 31.53 (1.36)**	
**Study area**	**16.08**	**2**	**< 0.001**	**SE: 22.36 (1.79)**^**a**^	**SW: 29.23 (1.48)**^**b**^	**N: 31.28 (1.27)**^**b**^
MP × sex	0.04	1	0.840			
MP × year	2.13	1	0.151			
MP × study area	0.85	2	0.653			

Different superscript letters indicate significant (*P* < 0.05) pairwise differences at Tukey *post-hoc* tests. Statistically significant predictors are highlighted in boldface

Differences between years were consistent with those described in previous paragraphs, with migration lasting longer and being slower in 2011 compared to 2010 (Table 7). Individuals from the SE study area also travelled faster and spent less time on migration than those from the other study areas (Table 7).

Individual LMP and MS of post-breeding migration did not correlate with those of pre-breeding migration (Pearson’s |r|≤ 0.09, n = 55 individuals, *P* ≥ 0.51). Similarly, there was no significant spatial consistency of migration routes, as the within-individual mean OWD between post- and pre-breeding migration routes (2.98 ± 0.21 SE, n = 55 individuals) was not significantly smaller than the mean among-individual OWD (2.99 ± 0.03 SE, n = 2970 comparisons; Pper_m_ = 0.94). Finally, at the population level, the mean OWD of post-breeding migration routes (2.14 ± 0.02 SE, n = 55 individuals and 2970 comparisons) was significantly smaller than the mean OWD of pre-breeding migration routes of the same set of individuals (2.59 ± 0.03, P_perm_ < 0.001). Overall, these results suggest a low degree of spatial consistency of migration tracks, and a greater variability of pre- versus post-breeding migration routes.

## Discussion

In the present study, we described the year-round movements of the largest sample analysed to date of barn swallows breeding in southern-central Europe. Our analysis revealed that these birds generally follow a clockwise loop migration pattern, moving through the central flyway during post-breeding (autumn) migration and then switching to the western flyway during pre-breeding (spring) migration, hence showing a tendency to avoid direct desert crossing during spring but not during autumn. In addition, our analyses showed that migration from the breeding sites to the non-breeding areas is more straight and less variable, though it lasts longer, compared to the opposite journey. The consistency of this pattern among individuals of different sexes and breeding in different sites, as well as between years, indicates that clockwise loop migration is a characteristic migration pattern among barn swallows breeding in south-central Europe.

Light-level geolocation often poses structural limits to the interpretation of the movement estimates of the tracked birds, limits that can lead to overconfident statements if the uncertainty of the process is not properly handled [63]. However, the specific migratory behaviour of the barn swallow, including long periods of residency and sudden short-duration, long-range and shade-free migration bursts, is more suited for geolocator analysis than other cases [64]. In addition, the careful pre-filtering of noisy twilight events we performed and the large sample size we considered, increases our confidence in the robustness of our reconstruction of migration trajectories and timing.

Our results are consistent with the increasing literature showing that loop migration is frequent among small passerine migrants (e.g. [12, 16, 19, 20]), including other barn swallow populations. Both barn swallows breeding in northern [15] and in central [22] Europe were indeed shown to follow different migration routes during a given migration cycle. What differs among these populations is the extent, the intra-population consistency, and the direction of the loop. On the one hand, northern populations show inter-annual and inter-individual consistency in migration routes similar to that reported here, but in the opposite direction (i.e. anticlockwise loop using the central flyway during post-breeding migration and the eastern one during the pre-breeding migration, [15]). On the other hand, central European populations breeding along the migratory divide use a mixed strategy spanning from clockwise to anticlockwise loop [22]. To date, among populations tracked with GLS so far, only birds from south-western Europe (i.e., the Iberian Peninsula) are known to use a single migration flyway in both directions [65], probably because their migratory journeys are shorter than those of the other populations and because their breeding sites are located along the European-African western flyway.

Why south-central European populations follow a longer route during pre- vs. post-breeding migration is open to speculation. A possibility is that suitable stopover sites are located in different places in each season (e.g. [9]. This can be especially important for species partly adopting a fly-while-foraging migration to overcome ecological barriers, such as raptors and likely also barn swallows [66]. Resource-mediated choice of migration routes in different seasons thus may result in the emergence of loop migration [9]. Hence, barn swallows from our study populations might perform a longer journey if the western flyway allows minimizing total energy and time costs compared to a straighter route. Indeed, long-distance flights might be particularly costly when birds must transport the heavy fuel load required for these flights. A westward detour may allow them to split the journey into a number of shorter steps requiring smaller fuel loads. Such a strategy might be more favourable to optimize migration efficiency than flying directly towards destination across wide ecological barriers, as in other bird species (e.g. [3, 67], but also in migrating bats [68]. Unfortunately, migration paths reconstructed from GLS are too coarse to allow a precise identification of stop-over sites *en route*.

While seasonal changes in resource distribution and abundance may be a critical aspect in determining migratory flyways, favourable winds can also have a profound influence on the transport economy of migrating birds. Indeed, selection of favourable tailwinds should improve migration performances, thus possibly contributing to the evolution of loop migration patterns (see e.g. [10, 11, 21]). For example, it is well-known that birds can set the timing of migration onset depending on tailwinds [69–71], but also select travelling altitudes that maximise wind support [72, 73]. Our findings are consistent with a previous study showing that optimal migration routes between sub-Saharan Africa and Europe are shifted westwards during pre-breeding compared to post-breeding migration because of wind regimes [74], hence favouring the direct crossing of the Mediterranean Sea and Sahara Desert without a detour during post-breeding migration. This is also the case for the western flyway [75]. This pattern may possibly explain why barn swallow perform a westward detour during pre-breeding migration, a pattern shared by most trans-Saharan migrants tracked to date (e.g. [18]. Yet, inter-individual spatial variation of pre-breeding migration routes was larger than that of post-breeding migration ones. Indeed, although the majority of individuals performed a westward detour during pre-breeding migration, entering Europe from the western Mediterranean, a non-negligible fraction of our birds migrated rather straight through the desert and arrived in Italy directly through the central Mediterranean (eastern Algeria, Tunisia, Libya).

Although pre-breeding migration was longer in terms of distances covered, it was also significantly faster than the post-breeding journey. Similar patterns have been repeatedly observed in birds, including barn swallows (reviews in [14, 47]. Proximately, a minimization of the total duration of spring migration can be explained by a much higher sustained flight speed during pre- vs. post-breeding migration, similar to that reported by previous studies of other species (e.g., ﻿[75–77]. In addition, a shorter stopover duration at sites during pre-breeding migration might have contributed to acceleration of pre- vs. post-breeding migration [78, 79].

From an evolutionary perspective, faster pre- than post-breeding migration could be expected because arriving early at the breeding site may have stronger fitness effects than arriving early at the non-breeding areas. Indeed, an early arrival to the reproductive areas is strictly related to increased annual fitness because birds have the possibility to (*i*) find suitable breeding sites and mates before competitors, (*ii*) mate faster, and (*iii*) temporally match their breeding activity with the peak of resources [80]. Indeed, early arriving birds have a larger reproductive success, both in males and females. This is the case also in the study population, whereby early arrival results in a larger seasonal egg production and fledging success [44, 46, 81, 82].

Since our analyses are based on individually tracked barn swallows, we could not only separate the migration features of different groups of individuals (sexes, years, geographical populations), but also investigate potential carry-over effects at the individual level. In this respect, our findings confirmed that the arrival date in the breeding grounds of an individual strictly depends on the departure date from the non-breeding areas ([40, 46], see also [83]), which in turn is highly correlated with arrival date to non-breeding areas. Such tight annual routines, typical of long-distance migrants [84], imply that the post-breeding period may have carry-over effects on reproduction and fitness. As expected, a straighter and faster pre-breeding migration was used by late-departing individuals, which allowed them to partly recover the initial delay [32]. However, such a deviation from the most frequent northward migration route may force them to use suboptimal corridors across large ecological barriers, potentially increasing mortality hazard. In addition, the use of suboptimal northward migration corridors might have increased the energetic costs of migration among late-departing individuals, with possible negative consequences for future reproduction and survival.

It is interesting to note that, although we observed a rather consistent pattern of migration routes at the population level, distance covered and migration speed showed a large variability between years and study areas (but not sexes), as well as among individuals depending on the moment when birds reach their non-breeding areas, when they start the northward migration, and on the location in Africa where they mainly spend their non-breeding period. Such differences, as well as the lack of any significant sex-related variation in time of migration and permanence in non-breeding areas, have been thoroughly discussed in a previous analysis of the same dataset [40]. The lack of sex differences in migratory behaviour is consistent with the weak sex differences in timing of migration and location of non-breeding areas reported in previous studies.

A final consideration concerns non-breeding itinerancy, which was also sporadically observed in other populations [22]. Notably, this vagrancy looks quite different from the progressive non-breeding seasonal movements of other trans-Saharan migrants and from those of Nearctic migrants, as it does not appear to be related to resource tracking. Indeed, it is performed by a small fraction of males only, which mostly move to the very south of Africa in a fashion resembling a prolonged migration (heading southward in autumn) rather than actual non-breeding vagrancy. These exceptional migratory routes, which look similar to those of north European populations (from e.g. Scandinavia or Great Britain) are most likely related to social attraction of migrating individuals, although it cannot be ruled out that they derive from individuals breeding in northern Italy or in southern Switzerland but immigrating from other populations [40].

In conclusion, we reported the results of the largest (in term of number of individuals tracked) analysis of migration routes of barn swallows, highlighting patterns of inter-individual and inter-seasonal variability, and confirming previous general knowledge of the migration routes of this species, including the lack of sex differences in migration behaviour. Yet, our study revealed novel details regarding inter-seasonal differences in migration features, as well as in migration routes and patterns of barrier crossing. Future studies linking migratory routes and fitness, as well as the analysis of the migratory behaviour of the same individuals during consecutive migration episodes, would provide a clearer picture of the ecological consequences of variable migratory decisions.

## Data Availability

The datasets used and/or analysed for this study are available from the corresponding author upon request.
